# A Review of Evidence on the Role of Digital Technology in Shaping Attention and Cognitive Control in Children

**DOI:** 10.3389/fpsyg.2021.611155

**Published:** 2021-02-24

**Authors:** Maria Vedechkina, Francesca Borgonovi

**Affiliations:** ^1^Faculty of Education, University of Cambridge, Cambridge, United Kingdom; ^2^Social Research Institute, Institute of Education, University College London, London, United Kingdom

**Keywords:** digital technologies, technology, attention, executive functions, children, television, videogames, multitasking

## Abstract

The role of digital technology in shaping attention and cognitive development has been at the centre of public discourse for decades. The current review presents findings from three main bodies of literature on the implications of technology use for attention and cognitive control: television, video games, and digital multitasking. The aim is to identify key lessons from prior research that are relevant for the current generation of digital users. In particular, the lack of scientific consensus on whether digital technologies are good or bad for children reflects that effects depend on users’ characteristics, the form digital technologies take, the circumstances in which use occurs and the interaction between the three factors. Some features of digital media may be particularly problematic, but only for certain users and only in certain contexts. Similarly, individual differences mediate how, when and why individuals use technology, as well as how much benefit or harm can be derived from its use. The finding emerging from the review on the large degree of heterogeneity in associations is especially relevant due to the rapid development and diffusion of a large number of different digital technologies and contents, and the increasing variety of user experiences. We discuss the importance of leveraging existing knowledge and integrating past research findings into a broader organizing framework in order to guide emerging technology-based research and practice. We end with a discussion of some of the challenges and unaddressed issues in the literature and propose directions for future research.

Socrates: ‘O most expert Theuth, one man can give birth to the elements of an art, but only another can judge how they can benefit or harm those who will use them. And now, since you are the father of writing, your affection for it has made you describe its effects as the opposite of what they really are. In fact, it will introduce forgetfulness into the soul of those who learn it: they will not practice using their memory because they will put their trust in writing […] and they will imagine that they have come to know much while for the most part they will know nothing […] since they will merely appear to be wise instead of really being so’. (Plato, Phaedrus)

## Introduction

In Phaedrus, Socrates warns about how writing might reduce people’s capacity to memorize information. Paradoxically, we know how Socrates felt only because one of his students, Plato, did not feel the same. As the brief passage indicates, the merits and pitfalls of new innovations have been debated at least ever since the invention of writing (Puchner, 2017).

Today, no one would question the importance of reading and writing and the fundamental role these skills have for individual developmental and for social progress. Yet, just as in the past Socrates worried about the pitfalls of writing because of its effect on memory, so many today worry about the impact digital technologies may have on attention and cognitive development. In the fact, many worry that new technologies, such as smartphones and tablets, may displace the amount of time individuals devote other useful activities. For example, the fact that pre-schoolers in the United States today are exposed to digital devices before they are introduced to books is taken as evidence that something problematic is happening to new generations (Hopkins et al., 2013). Similarly, the fact that on average, children spend anywhere from 7 to 9 h a day on digital technologies and media devices is a statistic that is intended to worry the general public about the excessive use of digital technologies, and the impact this might have on the minds and brains of young people (Rideout and Hamel, 2006; Uncapher et al., 2017).

Despite the often-sensational claims on the negative consequences of digital technology use for children’s cognitive development, the research literature provides a considerably more nuanced view. In particular, major brain changes or brain “rewiring” as a product of screen exposure, social media, or internet use is considered to be highly unlikely (Mills, 2014; Meshi et al., 2015; Loh and Kanai, 2016), and existing research identifies mixed results on the effects of technology on attention, cognitive control, and many other low-level and complex cognitive functions. The lack of scientific consensus on whether technology is good or bad for children suggests that effects may depend on circumstances: i.e., what is being used, by whom, how, when, with whom, and what outcome is considered.

The current review introduces and summarizes the available evidence from three main bodies of literature on the impact of technology on attention and executive functions, from both a functional and developmental perspective: television, video games, and digital multitasking with the aim of identifying key lessons for the current generation of digital users. We integrate research across developmental and educational psychology, and neuroscience to uncover the diverse theoretical camps, nuances, and contradictions within the existing scientific literature. We use existing research to propose an organizing framework which accounts for the role of content, context, purpose, individual and social factors in shaping observed outcomes in a way that is relevant to present-day digital users. A central theme emerges from the literature: the interaction between technology and human cognition is complex and multidimensional. We end with a discussion of some of the challenges and unaddressed issues in the literature and propose directions for future research to improve how technology-cognition interactions are studied, interpreted, and translated from research to practice.

### The Importance of the Early Years

This review focuses largely on implications that take place from the early years up to teen hood. This is primarily because most of the public interest in the role technologies play in shaping attention is targeted at children. Moreover, much of the existing research on adverse attentional outcomes of digital technology use concerns the developmental years. This is because the early and teenage years are a period in which experiences can have a disproportionate influence on later development (Kolb et al., 1998; DiPietro, 2000; Kolb and Gibb, 2011).

During the first years of life and up until early adulthood, the brain undergoes a period of significant plasticity, creating billions of new connections that are essential for the development of hearing, language, and executive skills (DiPietro, 2000; Fuhrmann et al., 2015). The childhood and the teenage years are therefore characterized as periods of important functional and structural reorganization, during which there is a high level of susceptibility of the brain to external stimuli.

The importance of the early years for cognitive development has led to concerns both in academic research as well as the popular media about the extent to which the use of different technologies during childhood and adolescence may have a lasting impact on brain development, as well as social, emotional, and cognitive functioning (Greenfield, 2003, 2015; Carr, 2011; Turkle, 2011; Alter, 2017; Orben and Przybylski, 2019). As more children access digital technologies sooner (Chaudron et al., 2018; Hooft Graafland, 2018), engage in a greater variety of activities using such technologies and in different contexts (Ofcom, 2020) the perceived importance of understanding the effects of technology use grows and guidelines designed to reduce harm and promote benefits proliferate (Straker et al., 2018).

### A Brief Overview of Attention and Cognitive Control

#### Attention

Attention is a limited cognitive resource which allows individuals to selectively filter the vast amount of information with which they are confronted at any given moment and prioritize certain elements while ignoring others (Carrasco, 2011). Attention is controlled by two separate but interrelated systems: Voluntary (top-down) attention is a controlled process and reflects past knowledge, goals, and expectancies, whereas involuntary (bottom-up) attention is automatic, reactive, and reflects sensory stimulation. The interaction between these two systems determines where and how individuals allocate attention in the surrounding environment (Corbetta and Shulman, 2002). Attention is also the gateway to higher-order cognition and determines how well individuals perform cognitively demanding tasks including reasoning, decision-making, and action-planning (Cowan, 2009; Diamond, 2013; Posner et al., 2014).

Attention is often presented as one of the central mechanisms through which digital technologies can interact with broader cognition. Research demonstrating a negative association between technology and attention is thus often used to assert the negative impact of technology on higher-level cognitive functions, like working memory, executive control, and learning; whereas literature showcasing a negative relationship between technology and learning often highlights attention as the main point of influence through which this effect occurs. Moreover, the appeal of attention over other constructs such as motivation (Ventura et al., 2013), self-regulation (Wei et al., 2012), and engagement (Rashid and Asghar, 2016), is that attention is more well-defined from an operational standpoint. For example, researchers can draw on behavioral analyses, cognitive measures, and physiological data as measures of attentional outcomes to understand the influence of screen-based media on attention.

#### Cognitive Control

Cognitive control, otherwise known as executive functions (EF) or executive control, is broadly defined as the cognitive processes that underlie motivation and goal-directed behaviors. EF are generally defined by three broad categories: *inhibition* (impulse and inhibitory control of automatic responses, self-regulation, and delay of gratification); *shifting* (task switching, mental-set shifting, and cognitive flexibility); and *updating* (working memory operations) (Dreher and Berman, 2002; Aron, 2008). Cognitive control is closely related to individuals’ voluntary and sustained-attention. It determines which information in the immediate environment will be attended and processed (Koechlin and Summerfield, 2007).

Executive functions are also related to higher-order cognitive functions and are predictive of a broad range of academic outcomes, including reading and numeracy (De Smedt et al., 2009; Alloway and Alloway, 2010; Fukuda et al., 2010; Diamond, 2013; Nouwens et al., 2017). The centrality of executive functions for broader cognitive functioning and learning has led to concerns that technology-use, particularly during sensitive periods of development, can interfere with young people’s cognitive control from both a transient (short-term) and developmental (long-term) perspective.

## Technology and Cognitive Outcomes: Exploring the Literature

Television and video games have historically been at the centre of public discourse and research on the effects of digital technology on developmental and cognitive outcomes. In today’s society, however, the growing use of ‘connected devices’ has led to a more generalized worry about the effects of screen-based devices on children (Bell et al., 2015).

This recent shift to a broader focus on ‘screen time’ in the research literature can be partially explained by the inability to differentiate between different forms of digital content (Orben and Przybylski, 2019). Connected and portable devices support an increasingly wide range of activities and contents. Mobile phones, for example, can be used to browse the internet, watch television, play games, and access social media. Traditional television, on the other hand, is being replaced by platforms like Netflix, Amazon Prime, and YouTube, which can be accessed using any device of choice (Ofcom, 2020). There is thus an inherent difficulty with isolating specific types of digital content to study despite an increasingly wide variety of digital content accessed by children, whereas grouping everything into broad categories like ‘screen time’, ‘mobile devices’, or ‘social media’ is of little use when attempting to draw precise conclusions about the cognitive implications of modern digital technologies.

Young people today are not just heavy users of digital devices but are also heavy practitioners of digital multitasking. The growing portability and early adoption of digital technology has meant that digital multitasking has become ubiquitous for the present generation of technology users. According to a recent survey by Pew Research Centre, 95% of teens have access to a smartphone, and 45% say they are online ‘almost constantly’ (Anderson and Jiang, 2018). Statistics such as these have led to mounting concerns about the impact of persistent digital multitasking on the brains and minds of today’s youth (Carlson, 2005; Rosen, 2010; Madden et al., 2013). Some scholars argue that frequent multitasking may be particularly detrimental for young people as it may interfere with the development of attention networks and executive functions, resulting in attention difficulties and a susceptibility for frequent task-switching over sustained attention (Levine et al., 2007; Fox et al., 2009). Others, however, argue that the early exposure and constant access to technology by today’s youth has created to a generation of ‘digital natives’, who have acquired a familiarity with technology and a multitasking proficiency quite unlike that of any previous generation (for review see: Prensky, 2001; Kirschner and De Bruyckere, 2017).

The rapid evolution of the technological landscape –the devices themselves, the nature of the content, and how they are used– has made it increasingly difficult to study how digital devices might interact with cognition in a way that is relevant to the present generation of media users (Marsh, 2014). This has created a lag in the scientific literature and also in the guidelines which they serve to inform (Straker et al., 2018). For example, despite social media being one of the main forms of content accessed through digital devices today, empirical research in the area remains scarce (Meshi et al., 2015), whereas most existing studies focus on Facebook, which is no longer as popular as other social media used by children and teens, such as Snapchat and Instagram (Ofcom, 2020).

The existing literature therefore largely reflects outdated devices and patterns of use, while also failing to take into account the nuances of how users engage with more modern ‘connected devices’. This is also reflected in policy guidelines which tend to focus on limiting the *quantity* of screen exposure, with little reference being made to the *quality* of engagement with digital content (American Academy of Pediatrics, 2016; Rütten and Preifer, 2016; Ponti et al., 2017). However, there is still much value to be gained from the existing literature if adequately put in context. In particular, the building blocks that have been identified in past literature can provide useful insight for evaluating how newer and more complex forms of digital media might interact with human cognition.

Furthermore, given the growing variety of digital technologies, it becomes increasingly important to identify whether effects on cognition differ in systematic ways depending on the context in which digital media are used, their content and user characteristics. Doing so requires identifying and testing moderators of underlying relationships between digital technology and cognition. This review examines the existing literature to identify which factors appear to consistently moderate differences in the strength of the association between technology use and cognition. This information can help formulate hypotheses to guide future research and increase the likelihood that such studies will inform evidence-based policy to benefit the current generation of digital users.

Television and video games have been favorite leisure time activities among children worldwide for decades. The current review covers the television and video game literature as these two forms of technology they have been extensively studied in the past and because many of the features characterizing television and video games constitute the building blocks of more modern digital content on the internet and social media. We then review the newer digital multitasking literature, which addresses some of the contextual considerations which are most relevant today: technology is often used in combination with other activities, including work, school, and social interaction. Indeed, there is an additional challenge associated with studying the cognitive implications of modern digital devices in that they can be accessed anywhere and anytime.

### Television

As the form of media that has been around longest, television has historically been the focus of much research on the influence of technology on cognition and development. The experience of viewing television has remained fairly constant throughout the years, although different, more modern, forms of digital media afford users greater opportunity to control what, when and how they view video content. Nonetheless, many of the changes that newer forms of digital media afford could also be achieved in the past by augmenting the limited possibilities for active viewer control through ad-on technologies, such as video recorders, DVD players and antennae. As a result, there is a relatively large literature base exploring television and cognitive outcomes in children, and many of the features that characterize modern-day viewing of video content have already been explored in the television literature. However, the quality of much of the literature is low, offering mainly correlational and cross-sectional evidence with small effect sizes, thus leading to identification problems. Moreover, the existing literature is ripe with inconsistencies and conflicting results, a possible indication of heterogeneous treatment effects: results could differ, for example, depending on the type of television programming considered, population group studied, and cognitive outcomes measured, factors that are not always possible to examine and account for when considering estimated associations.

#### Attention Engagement During Television Viewing

Television programming is characterized by features such as fast-paced images, highly salient stimuli, and, in many cases, content breaks (e.g., commercials). Some scholars have argued that such features may not be conducive to the development of cognitive and attentional control in children. There remains, however, some disagreement as to the exact mechanisms through which these formal features might interact with cognition in both the short- and long-term.

Anderson et al. (1987) first coined the term *attentional inertia* to describe the phenomenon that children become progressively less likely to look away from television after they had been watching for some time. Based on their observations, the probability of a child looking away peaks at around 1 second of viewing and then progressively decreases with time, leveling off at the 15-second mark (Anderson et al., 1987; Burns and Anderson, 1993). Subsequent studies also found that viewers are less likely to react to distractors or changes in content if they had been watching television for at least 15 seconds (Anderson and Lorch, 1983; Anderson et al., 1987; Richards and Turner, 2001). These findings led scholars to theorize that viewers initially attend programming based on whether the content is comprehensible, however, after some time, attention becomes generalized to the medium (television screen), rather than the content, so that breaks and changes in content do not necessarily distract away from the screen, and may actually have the opposing effect (Anderson et al., 1987). This view led scholars to consider that television programming encourages passive viewer engagement, prioritizes bottom-up processing, and therefore does little to train children’s sustained attention (Lillard and Peterson, 2011).

Other studies have shown that content that is difficult to understand or filled with breaks and distractions results in the continuous disruption of sustained attention (Lorch and Castle, 1997; Pempek et al., 2010; Richards, 2010). This has led some scholars to argue that certain features of programming, like shorter scene lengths, and content cuts, may overstimulate developing brains and can be especially detrimental to the development of cognitive control in children (Wright and Huston, 1983; Valkenburg and Vroone, 2004; Goodrich et al., 2009). Indeed, several cross-sectional studies found that preschool viewing of quality programming *without* commercials was positively associated with measures of attention and executive control, whereas viewing similar content *with* commercials correlated with poor performance (Hudon et al., 2013; Nathanson et al., 2014). These findings suggest that the presence of commercial breaks require children to constantly disengage and re-engage their attention to the screen, promoting a reactive style of attention and making it particularly difficult for young viewers to link concepts together and extract meaning from programming (Valkenburg and Vroone, 2004). This is especially problematic given that modern web-based video content is often free of charge when advertisements are introduced, requiring user subscriptions to avoid commercial content (Radesky et al., 2020), which in the long term could lead to increased socio-economic (SES) disparities in attention as a result.

The experimental literature also suggests that the pace at which content is displayed can be detrimental to children’s sustained attention. Lillard and Peterson (2011), for example, reported that just nine minutes of exposure to a fast-paced cartoon impaired children’s subsequent performance on a task measuring cognitive control. Rapid sequencing has been shown to capture attention in a more automatic fashion, with decreased involvement from prefrontal cortices which are responsible for effortful attention allocation (Buschman and Miller, 2007). This view posits that children become passive recipients of television content whose attention to the screen is maintained through perceptually rapid sequencing and salient audio-visual stimuli (McCollum and Bryant, 2003). These salient features repeatedly orient and maintain children’s otherwise distractable attention to on-screen change. Some scholars suggest that this results in a reliance on the environment to maintain attention engagement, thereby prioritizing bottom-up processing biases and leading to distractibility during other everyday tasks (Kostyrka-Allchorne et al., 2019). From this perspective, the effort to encode fast-paced non-normative television content could tax children’s cognitive resources in the short term (Lang et al., 1999), and do little to train more effortful attentional control in the long term (Lillard and Peterson, 2011).

Brain imaging studies and experimental evidence on how video content influences children’s cognitive and neural function, however, remain scarce leaving researchers to hypothesize the underlying processes for observed effects and making the directionality and causality of measured outcomes difficult to ascertain (Takeuchi et al., 2016). Moreover, movie and television content has changed dramatically over the last few decades. For example, technological advances in the industry have led to changes in video style characterized by shorter shot durations, enhanced motion and greater luminance changes, in an attempt to increase viewer engagement over longer periods of time (Cutting, 2016). The growing use of computer animation in child-directed content since the 80s has also led to an increase in the average frames per second to 75–120, compared to 15 frames in traditional hand-drawn cartoons (Rick, 2012).

#### Television Viewing During Infancy and Later Attention Difficulties

The notion that certain features of television programming can negatively interact with the mechanisms required for the top-down control of attention has led to concerns that television viewing during infancy could lead to later attention difficulties. Indeed, many longitudinal and cross-sectional studies have linked television viewing during infancy with adverse cognitive outcomes in later childhood (Özmert et al., 2002; Zimmerman and Christakis, 2007; Barr et al., 2010). For example, one much-cited longitudinal study using data from the 1979 National Longitudinal Youth Survey (United States), reported that early television exposure (at ages 1 and 3) was significantly associated with attention problems at age 7 (Christakis et al., 2004). Follow-up evidence indicated that each additional hour of daily television before age 3 was associated with a linear decrease in reading and attention scores, and an increase in risk for ADHD (Zimmerman and Christakis, 2005).

However, while these studies appear to suggest that early television viewing may increase the risk of developing attention difficulties, the weight of the evidence does not conclusively support a clear association between television viewing and adverse cognitive outcomes. Indeed, several studies have failed to replicate the association between television viewing in moderate amounts and the development of later attention difficulties. For example, subsequent reanalysis of the National Longitudinal Youth Survey dataset using a non-linear model found that the risk of developing attention difficulties was significant only for 10% of children who watched over 7 h of daily television (Foster and Watkins, 2010), indicating that television exposure during infancy may be detrimental to attention only at very high levels of viewing.

In support of this interpretation, Obel et al. (2004) used a similar design with a Danish sample, found no significant association between early childhood television exposure and the development of attention difficulties later on. The authors attributed these conflicting results to the fact that Danish children watch less television on average than their American counterparts (only 6% of Danish children watched over 2 h of daily television at 3 years, compared to 50% in the American sample). Therefore, it is likely that Danish children, on average, do not surpass the critical daily viewing threshold where the association between television viewing and attention may become significant (Obel et al., 2004).

There are also important generational changes in the way users watch television, which are often overlooked in the literature. Many of the longitudinal studies, for example, rely on data collected from different birth cohorts ranging from the 1970s to present-day and may therefore differ along several characteristics pertaining to individual children and their families, to the context and social environment in which they operate and the content of the television programs being watched (Christakis et al., 2004; Zimmerman and Christakis, 2005, 2007; Stevens and Mulsow, 2006; Landhuis et al., 2007).

Although television remains the most frequency-used device for viewing video content, over the last decade, there has been a steady decline in broadcast television child viewership on traditional television sets. The growing use of streaming services and platforms such as YouTube, Hulu and Netflix may partially account for the annual surge of 49% in tablet use among 3–4 year-olds in the United Kingdom (Ofcom, 2020). Moreover, the transportability, simple user interface and perceived educational value of touch-screen devices means that parents are more likely to use them as part of their daily routine with young children (Siibak and Nevski, 2019; Ofcom, 2020). To increase our understanding of how video content may be impacting cognitive function in the present generation of young people, additional research is needed to discern whether viewing video content through portable media significantly differs from viewing through traditional television sets.

#### The What, When, and How: The Importance of Television Quality

By the age of three (in contrast to early infancy), children develop the ability to attend, comprehend, and therefore, learn from age-appropriate television content (Anderson and Hanson, 2010; Pempek et al., 2010). Indeed, findings on the impact of television viewing in late childhood and adolescence on attention and higher-order cognition are inconsistent (Johnson et al., 2007; Landhuis et al., 2007; Parkes et al., 2013). Such inconsistencies have been considered to result from an important role of the quality of television programming (Anderson et al., 2001; Zill, 2001; Kostyrka-Allchorne et al., 2017).

Many studies suggest that the *type* of programming may be more important than the total viewing time and that any association between television and cognition is dependent on both the content *and* context of viewing (for review see: Zimmerman and Christakis, 2007; Kostyrka-Allchorne et al., 2017). For example, Zimmerman and Christakis (2007) reported that viewing entertainment television before age 3 was associated with adverse attention outcomes later on. However, the same study found that viewing educational programming was not associated with any adverse cognitive outcomes. Indeed, childhood viewing of educational television may be beneficial to the development of executive functions, basic academic skills, and social behavior in children over two (Wright et al., 2001; Zill, 2001; Fisch et al., 2005; Schmidt and Anderson, 2009; Anderson and Subrahmanyam, 2017; Kostyrka-Allchorne et al., 2017).

What qualifies as ‘high-quality’ or ‘educational’ content, however, has been another topic of contention among researchers. Some studies have linked educational cartoons like *Blues Clues* and *Dora the Explorer* to positive learning outcomes and executive function in children (Linebarger and Walker, 2005; Barr et al., 2010; Fisch, 2014). Others, however, find that exposure to any type of content that provides non normative stimulation, characterized by rapid pace and atypical sequencing, can have negative consequences for the development of attention and cognitive control, even if the storyline itself is educational (Goodrich et al., 2009; Christakis et al., 2012; Nathanson et al., 2014). Moreover, recent research by Kostyrka-Allchorne et al. (2019) suggests that the degree of realism of video content may affect young children’s executive function to a greater degree than the pace of programming, and that attention is sensitive to the interactive effects between realism and pace. The authors suggest that the degree of realism of television programming may provide a buffer against the negative effects of rapid pace.

Another theory posits that there is an opportunity cost to watching television. Proponents of this view argue that irrespective of the absolute effect of viewing entertainment programs on television on attention during sensitive periods of development, watching such programs will be associated with worse outcomes because it will reduce the amount of time devoted to more enriching activities (Mutz et al., 1993; Kuhl et al., 2003; Anderson and Pempek, 2005). Indeed, even exposure to background television has been shown to disrupt sustained toy play and reduce the quality and quantity of parent-child interactions, which is critical for language acquisition and the development of cognitive and social skills (Schmidt and Vandewater, 2008; Christakis et al., 2009; Kirkorian et al., 2009; Barr et al., 2010; Pempek et al., 2014).

The growing availability of digital content that can be consumed as and when users decide may be increasing the time children spend accessing video content, particularly via small-screen devices (Ofcom, 2020). Conversely, average daily television time via traditional TV sets has been steadily decreasing year on year among children in the United Kingdom (Ofcom, 2020). Importantly, preliminary evidence indicates that the medium through which video content is delivered may matter. For example, infants may learn more readily from touch screen devices than through traditional television screens (Kirkorian et al., 2016). Touch screen devices are interactive and therefore allow children to control the speed and flow of information, which may increase engagement and ability to learn from video content. As technologies evolve, their ability to engage and stimulate children will also change, thereby requiring researchers to re-evaluate mode-of-delivery effects.

#### The Who, Where, and Why: Individual Characteristics, Social, and Family Factors

The opportunity cost hypothesis of early television viewing is supported by evidence that the relationship between television viewing and cognitive outcomes seems to differ by social and family factors (Wright et al., 2001; Linebarger et al., 2014). Indeed, studies that have taken into account relevant demographic factors often report that the link between television and cognitive outcomes all but disappears once household characteristics are accounted for Foster and Watkins (2010), Linebarger et al. (2014). Moreover, parenting style and family environment seem to moderate the relationship between television and cognitive outcomes by determining the value of alternative uses of children’s time and the type of content being accessed.

Research indicating that the implications of television viewing depend on the type of activities it displaces is relevant for understanding why the effect of television can be highly heterogeneous. Television viewing is associated with worse outcomes for children whose alternative use of time would be high-quality interaction with their parents, but more positive outcomes among groups of children who lack such experiences (Comstock and Paik, 1991; Linebarger et al., 2014). For example, among low-income, low-educational attainment or immigrant (e.g., non-native speaking) households, educational programming is associated with positive educational outcomes, such as language development and executive function enhancement (Linebarger et al., 2014). Education television may therefore be particularly beneficial for underprivileged children and for bridging academic gaps between different socio-economic backgrounds.

In contrast, there is also evidence that early television viewing habits may exacerbate disparities in cognitive performance between high-SES and low-SES children because parental resources determine the content that is being accessed (Zimmerman and Christakis, 2005; Ponti et al., 2017). Parents with fewer financial resources and who are less involved in their children’s daily activities, tend to spend less time curating television content for their children (Ponti et al., 2017). Moreover, children with parents with higher educational qualifications tend to watch less television overall because of the high quality and engaging nature of leisure time activities (Truglio et al., 1996; Certain and Kahn, 2002; Rideout and Hamel, 2006). At the same time, when they do watch television, children with more educated parents are more likely to do so with their parents (co-viewing), which may result in a more cognitively enriching viewing experience overall. Parents who pose questions and provide explanations of the material being watched can support children’s ability to learn from it (Barr et al., 2008). The available evidence on the cognitive benefits associated with co-viewing, however, remains inconclusive as there currently lacks compelling support for any benefits beyond that of increased child-parent interaction, independent of the content of the activity (Lee et al., 2017).

Finally, it should be noted that a large body of literature concerns the relationship between screen-based media, particularly television, and ADHD in children. The weight of the evidence suggests that any association existing between electronic media and ADHD is complex. Longitudinal research supports that there may be a negative (albeit small) association between television viewing and ADHD (Acevedo-Polakovich et al., 2007). However, as most studies are correlational and cross-sectional, the causality of this association remains unclear (for reviews see: Nikkelen et al., 2014; Beyens et al., 2018). For example, it is possible that children with ADHD simply prefer to watch more television due to their cognitive-behavioral dispositions (e.g., higher thresholds for engagement and a preference for highly stimulating content (Beyens et al., 2018). Moreover, children with ADHD are more likely to watch television with their parents, perhaps because television is seen as a low-stress activity to do together and can serve as a substitute for social interaction (Acevedo-Polakovich et al., 2007; Schmidt and Vandewater, 2008). However, as ADHD is a medical condition, rather than a normal variation in cognitive faculties, a thorough analysis of the related literature is beyond the scope of the present review.

#### Television Summary

Taken together, the existing evidence exploring the cognitive implications of childhood television viewing remains inconclusive. Certain features of television programming, like fast-pace and non-normative stimulation, may be harmful to very young children by taxing cognitive resources and encouraging bottom-up processing (Valkenburg and Vroone, 2004; Goodrich et al., 2009; Lillard and Peterson, 2011). However, strong evidence supporting any long-term association between television viewing in moderate amounts and cognitive development is currently lacking. Moreover, the literature suggests that the quality of content and social context of viewing are important moderators of the association between television and cognition in children. As children develop the ability to comprehend, and therefore, *learn from* television content (Anderson and Hanson, 2010), the long-term cognitive implications may differ depending on whether the alternative uses of children’s time would be devoted to more enriching activities, such as learning or high-quality interaction with parents (Linebarger et al., 2014). Educational television programming may, therefore, be particularly beneficial for improving long-term academic outcomes for children from non-native speaking households and lower socio-economic backgrounds.

### Video Games

Video games are electronic games that require behavioral interaction with a user interface in order to generate audio-visual feedback on a display device. Games are broadly classified based on the system they are played on: arcade games, consoles, handheld devices, computers, and more recently, mobile phone devices. They also encompass various genres based on game-play type and purpose: action, shooter, adventure, role-playing, simulation, strategy, puzzles, cards, racing, and educational (Adams, 2014). Contrary to viewing video content, which affords users only limited possibilities for active engagement, video games are interactive activities that encourage players’ cognitive and motor engagement with simulated worlds (Shaffer et al., 2005).

With the growing ease of access and popularity of video games over the last few decades, there have been mounting concerns over the cognitive, behavioral and developmental implications of gaming (Elson and Ferguson, 2014; Ofcom, 2020). At the same time, a burgeoning area of research has emerged which focuses on investigating if, and to what extent, video games are associated with positive outcomes and whether they could be harnessed to enhance various cognitive functions in both children and adults (Powers et al., 2013).

In contrast to television studies, however, video game research conducted on younger children is only beginning to emerge. Most of the existing literature thus tends to focus on adult or adolescent populations. This is in part a reflection of the fact that, in the past, playing videogames required physically entering arcades, and in part of the fact that young children are unable to properly engage with complex video games (e.g., action video games) that are often the focus of academic research.

#### Attention and the Brain During Video-Game Play: Evidence From Training Studies

Video games are characterized by similar formal features to television programming, including rapid image succession and highly salient stimuli (Swing et al., 2010). However, in contrast to the overly negative focus of the television literature, the gaming literature is somewhat more nuanced, perhaps prompted by the well-documented developmental benefits of everyday play in young children (Shaheen, 2014), and the active engagement during video-game play. For example, the cognitive benefits of video-gaming and the use of game-based cognitive training tools have been widely investigated from both a theoretical and empirical perspective, particularly in older populations (Feng and Spence, 2018).

The potential use of video games as tools for cognitive enhancement has been a topic of much investigation in the gaming literature. Some scholars suggest that video games provide ideal learning opportunities for children by promoting informal exploratory learning and enhancing problem-solving skills (Greenfield et al., 1994). Players must continuously integrate a range of sensory inputs, respond (or ignore) perceptually salient stimuli and implement adaptive strategies to meet the ever-changing demands of complex virtual environments (Bavelier et al., 2012a). Proponents of the *Learning to Learn Hypothesis* argue that gaming can be used to enhance broad aspects of cognition, and can lead to general improvements in attentional capacity, cognitive control, pattern recognition, problem-solving abilities, and more efficient learning strategies (Bavelier et al., 2012a; Green and Bavelier, 2012; Prensky, 2012).

Numerous training studies have demonstrated improvements on *specific* measures of visuospatial cognition after short periods of playing video games, including visuospatial selective attention (Feng et al., 2007; Spence et al., 2009), visual search (Green and Bavelier, 2003, 2007; Wu and Spence, 2013), visuospatial working memory (Thorell et al., 2009), response selection (Dye et al., 2009a,b; Hutchinson et al., 2016), multiple object tracking (Oei and Patterson, 2013, 2015), dual-task switching (Li et al., 2009; Strobach et al., 2012) and spatial reasoning skills (De Lisi and Wolford, 2002; Sims and Mayer, 2002; Haier et al., 2009; Boot et al., 2011; Uttal et al., 2013). These findings are further supported by evidence that playing action video games, even over a relatively short period of time, can modify the neural responses associated with top-down cognitive control and improve the modulation of visuospatial selective attention (Bavelier et al., 2012b; Krishnan et al., 2013).

The weight of the evidence from gaming literature, however, suggests that that the degree of cognitive transfer is largely dependent on the content of the game and on the specific cognitive skills that are recruited during game-play (Green and Bavelier, 2006; Boot et al., 2008; Strobach et al., 2012; Oei and Patterson, 2014, 2015; for review see: Subrahmanyam and Renukarya, 2015). In other words, it seems unlikely that video-game play can lead to *broad* improvements in cognitive function on tasks which are markedly different from that in which the original training occurred (Pillay, 2003; Subrahmanyam and Renukarya, 2015). Neural evidence also suggests that skill transfer is more likely to occur between overlapping brain regions (Dahlin et al., 2008), supporting that transfer is limited where a game and task do not recruit similar perceptual templates, and that any post-training benefits of gaming are likely task-specific (Przybylski and Wang, 2016; Azizi et al., 2017; Sala and Gobet, 2018).

Moreover, although cognitive improvements have been reported across numerous video game training studies (Feng et al., 2007; Dye et al., 2009a; Li et al., 2009; Spence et al., 2009), many other studies either do not support, or directly contradict, the visuospatial and perceptual benefits of gaming (Boot et al., 2008, 2011; Kennedy et al., 2011). Indeed, the evidence on the skill benefits of gaming topic is inconsistent and is characterized by an overreliance on self-report, small sample sizes, and cross-sectional study design. Additionally, much of the experimental evidence in the domain has been collected using convenience samples of adults, or intervention studies on university students, and therefore suffers from a plethora of methodological shortcomings which limits the generalizability of findings (Boot et al., 2011; Przybylski and Wang, 2016).

#### Cognitive Profiles of Video-Gamers

In recent years, there has been mounting interest in determining whether video-game play during development is associated with benefits or deficits to long term cognitive outcomes. For example, several studies have reported associations between video-game play during childhood and later attention difficulties (Swing et al., 2010; Gentile et al., 2012). Some have argued that this relation could be bidirectional: Children who suffer from attention problems may be more likely to spend time engaging with video games, which in turn can further interact with their cognitive capacity (Gentile et al., 2012). However, longitudinal evidence which examined the association between video game exposure during childhood and attention difficulties in early adolescence identified an association even after controlling for earlier childhood exposure (Swing et al., 2010), suggesting that the link between gaming and attention may not simply be reduced to pre-existing group differences. Moreover, the association between gaming and attention across different age groups supports the possibility of long-lasting and cumulative consequences of intense gaming exposure (Swing et al., 2010).

However, not all research supports that playing video games during childhood is detrimental to attention, especially in moderate amounts. One longitudinal study, for example, found that in contrast to early television viewing, playing electronic games at age 5 was not significantly associated with any adverse behavioral or cognitive outcomes at age 7 (Parkes et al., 2013). Another study of 7 to 11-year-old children found that only children who played over 9 h of video games per week were more at risk of conduct problems and reduced prosocial abilities. Moreover, moderate gaming frequency (1 h per week) was associated with *superior* visuomotor skills in the same sample (Pujol et al., 2016).

There is also evidence indicating superior cognitive control among action video-game players as young as 7 years old (Dye and Bavelier, 2004; Wu and Spence, 2013; Cain et al., 2014). For example, experienced action video-game players seem to be better than non-gamers at ignoring irrelevant distractions (Green and Bavelier, 2003; West et al., 2008; Dye and Bavelier, 2010; Mishra et al., 2011; Chisholm and Kingstone, 2012, 2015), attending information over long periods of time (Boot et al., 2008), localizing targets (Chisholm et al., 2010; Hubert-Wallander et al., 2011; Wu and Spence, 2013), tracking multiple objects simultaneously (Green and Bavelier, 2006; Bavelier et al., 2010), and switching between tasks (Strobach et al., 2012; Pohl et al., 2014; Bavelier and Green, 2019). One study by Colzato et al. (2013) found that experienced gamers were faster and more accurate on working memory updating and monitoring (N-back and stop-signal tasks), but showed comparable response inhibition to non-gamers. These results suggest that video games might enhance cognitive control without necessarily affecting impulsivity, which is in direct contrast to the much-cited reports linking video games to attention difficulties and violent tendencies in children (Barlett et al., 2009; Gentile et al., 2012).

There is also evidence to suggest that playing video games (particularly action video games) can enhance specific cognitive skills in the long-term (for reviews see: Spence and Feng, 2010; Bavelier et al., 2012a; Green et al., 2016). Cross-sectional studies report that frequent gamers, compared to non-gamers, exhibit superior attentional capacity in central and peripheral vision processing (Green and Bavelier, 2003; Dye and Bavelier, 2004), performance on a variety of visuospatial tasks (Green and Bavelier, 2006, 2007; Hubert-Wallander et al., 2011; Wu and Spence, 2013; Green et al., 2016). Consistent with behavioral findings, brain imaging studies suggest that experienced gamers exhibit less activation in the visual cortical area for motion processing and in the frontoparietal network during attention-demanding tasks, suggesting better top-down control of visuospatial selective attention (Bavelier et al., 2012b; Feng and Spence, 2018). Therefore, although mostly based on cross-sectional data, the literature reveals that there may be long-term cognitive benefits to playing certain video games that require a high degree of user engagement, particularly in older age groups.

While boys have historically dominated the gaming sector, the growing popularity of online games among young people has led to a shift in the gender gap: the proportion of girls in the United Kingdom playing video games online has increased from 39% in 2019 to 48% in 2019 (Ofcom, 2020). Indeed, mobile gaming is currently the fastest growing video game segment, becoming increasingly popular with young people, among whom mobile games are the second most downloaded type of app in 2019 (Mordor Intelligence, 2020). Despite this growing popularity of video games across different demographics, there still remains much debate regarding whether the reported benefits of game-play are due to pre-existing group differences between gamers and non-gamers, and whether extensive video game practice by non-gamers can lead to cognitive benefits and performance improvements on unrelated tasks.

#### The Who and Why: Individual Differences and Other Considerations

Individual differences in cognitive capacity, motivation, engagement, interest, or even specific subtypes of games individuals engage in could be driving the effects reported in small-scale studies looking at the cognitive implications of gaming (Boot et al., 2008; Przybylski and Wang, 2016). As is true for learning in general, some individuals acquire certain skills faster than others. For example, several brain imaging studies have shown that neuroanatomical variation in regional brain volume correlate with differences in skill acquisition after playing a video game (Erickson et al., 2010; Basak et al., 2011). Any training benefits derived from gaming may, therefore, also depend on players’ latent potential for improvement (Bavelier and Green, 2019).

The behavioral and the neural literatures also suggest that prior gaming experience may account for some of the observed variance in skill acquisition, performance improvement, and skill transfer rates (Spence et al., 2009; Wu et al., 2012; Bavelier and Green, 2019). For example, many scholars argue that the widely disputed gender differences in visuospatial attention may be due to the fact that action video games predominantly attract male audiences (Feng et al., 2007; Dye et al., 2009b) and that when selection effects are eliminated, playing an action video games can reduce gender disparities in both spatial attention and mental rotation ability, with women benefiting more than men from the training (Feng et al., 2007). Moreover, several training studies have demonstrated that individuals with the poorest baseline attention performance generally benefit the most from video-game training – an indication of decreasing marginal returns to gaming (De Lisi and Wolford, 2002; Whitlock et al., 2012).

The perceptual benefits of game-play may also vary as a function of age (Dye et al., 2009b; Hartanto et al., 2016). This is because different components of attention develop at different rates. Attentional orienting and executive control, for example, are stable by age 7, whereas attentional alerting continues to develop well into adolescence (Rueda et al., 2005). In support of this, performance enhancements as a result of video-game training seem to decline with age, with adolescents benefiting more than adults, and young children benefiting most (Lövdén et al., 2010; Hartanto et al., 2016). Similarly, cross-sectional studies have shown that individuals who begin playing video games before the age of 10 perform better on various measures of attention, compared to those who began playing at later ages, a possible indication of critical-age effects (Dye et al., 2009b; Latham et al., 2013).

The age of onset at which individuals first begin playing video games is, therefore, an important factor to consider: the earlier the initial age of onset and the longer the lifelong gaming practice, the greater the cognitive interaction (Hartanto et al., 2016). As such, the common operationalisation of video-game expertise based on the frequency of weekly gameplay (Bavelier et al., 2012b), without accounting for differences in cognitive plasticity across different stages of development, may not adequately capture these crucial age considerations (Hartanto et al., 2016).

#### The What, When, and How: Video-Gaming Beyond the Lab

It may be premature to discredit altogether that the gaming may confer broad cognitive benefits, beyond specific experimental measures of cognitive ability. Some studies, for example, have linked gaming to superior task persistence and motivation (Przybylski et al., 2010; Ventura et al., 2013), as well as to self-reported improvements in problem-solving abilities which tend to predict better academic performance (Adachi and Willoughby, 2013).

Brain imaging studies also support that there may be some cognitive benefit to video-game play (Kühn et al., 2011; Pujol et al., 2016). One study, for example, found that frequent gamers showed increased functional connectivity in the basal ganglia, a region associated with a variety of functions including procedural learning and skill acquisition (Pujol et al., 2016). This view is further supported by studies showing that playing video games stimulates the neural reward system, which is associated with learning and positive reinforcement (Koepp et al., 1998; Weinstein and Lejoyeux, 2015).

Video games may also facilitate task-specific learning by providing continuous feedback and clearly defined goals, which is thought to increase arousal, attention, and motivation during tasks (Przybylski et al., 2010; Feng and Spence, 2018). These features may provide the optimal learning environment for children with ADHD (Schmidt and Vandewater, 2008). For example, studies of individuals with ADHD often rely on computer game tasks for studying cognitive performance in this group (Houghton et al., 2004).

Conversely, some scholars argue that frequent gaming may increase dependency on external rewards (Swing et al., 2010). For example, one study found that frequent teenage gamers showed stronger functional activation in the ventral striatum during loss processing, a region associated with dopaminergic responses to feedback anticipation and reward processing (Kühn et al., 2011). However, as neuroimaging studies rely on small samples and do not account for prior group differences, the causal direction of the association between gaming and neurocognitive outcomes remains unclear.

As with television, the form and purpose of a game may be an important factor to consider when assessing the cognitive implications of video-gaming. For example, preliminary research suggests that educational games presented through age-appropriate *interactive* mediums (e.g., a touch screen device) may support literacy and mathematics skill acquisition for young children, particularly for those from underprivileged households (Neumann and Neumann, 2017; Neumann, 2018). These results suggest that as digital technologies become more sophisticated and allow for more immersive and interactive experiences, the cognitive benefits of gaming may become more pronounced.

#### Video Games Summary

Taken together, the evidence summarized suggests that gaming may be associated with both positive and negative outcomes which depend on the intensity of gaming, type of game, the outcomes being measured and individual characteristics of players. Action video games, in particular, seem to be associated with benefits in visual and spatial selective attention in older populations, particularly on tasks requiring top-down cognitive control. However, strong evidence supporting broad skill enhancement beyond specifically trained tasks is currently lacking, and only few studies have been conducted on children. Moreover, the literature suggests that individual differences in cognitive function and prior gaming experience may, at least in part, account for the reported differences between gamers and non-gamers, further supporting a bidirectional link between gaming and cognition (Powers et al., 2013). In other words, individuals may be more drawn to video games (and other digital content) that suit their own abilities. Due to differences in cognitive plasticity across different stages in development, the age of onset at which individuals first begin playing video games is also an important factor to consider: the longer the lifelong gaming practice, the greater the cognitive implications (Hartanto et al., 2016). Finally, while there are preliminary studies supporting the motivational benefits of interactive technologies, there is a paucity of research on whether the purpose of a game (e.g., academic), context (e.g., school) and family factors (e.g., SES) may moderate long-term cognitive outcomes of video-game play.

### Media Multitasking

In contrast to traditional digital technologies like television and video-game consoles not connected to the internet, which allowed users to perform only one or a limited set of activities, newer digital devices can be accessed anytime, anywhere, and while performing multiple concurrent tasks. In other words, the line between being ‘online’ or ‘offline’ is becoming increasingly blurred. Moreover, the same device can be used to play video games; search for information; talk with friends; upload pictures on social media and watch videos. These trends highlight the growing difficulty with isolating when, how, and why the current generation is using their digital devices. The media multitasking literature accounts for some of these contextual considerations which are most relevant to the current generation of digital users by emphasizing that using technology while performing other tasks, or engaging in different activities with the same medium can moderate the cognitive outcomes associated with its use.

#### Mechanisms of Multitasking

Multitasking is defined as the simultaneous processing or execution of two or more tasks. The behavioral and neurocognitive literature suggests that multitasking is, in fact, just rapid task-switching. This means that tasks are processed in succession (rather than simultaneously) resulting in limited attentional resources being shared between two or more individual tasks (Foerde et al., 2006; Colom et al., 2010). Such task-switching behavior may place increasing demands on neurocognitive networks that are responsible for controlling and sustaining attention (Rubinstein et al., 2001; Alzahabi and Becker, 2013; Waskom et al., 2014).

One way the effects of multitasking are examined in the scientific literature is by analyzing ‘task switch-costs’, which are reductions in performance speed or accuracy resulting from task-switching. When individuals switch from one task to another, the benefits of automaticity and efficiency relating to the former task are lost, and additional effort is required to undertake the new task (Braver et al., 2003; Waskom et al., 2014). There is a large body of evidence documenting the performance deficits associated with task-switching, suggesting that trying to carry out a number of tasks simultaneously is generally not more efficient than completing a single task at a time (Corbetta and Shulman, 2002; Jeong and Hwang, 2016; Kirschner and De Bruyckere, 2017). Moreover, task-switching behavior often occurs automatically such that individuals tend to underestimate their task-switching frequency and associated performance deficits (Brasel and Gips, 2011).

The ease with which individuals multitask is determined by both the amount (quantity), as well as by the type (quality) of cognitive resources required for carrying out a given combination of tasks. As the complexity of a task increases, so does the cognitive workload needed to maintain performance on that task (Smith et al., 2001; Wickens, 2008). However, as certain activities become automatised with practice, less cognitive effort is required to carry them out, which may free up resources for the simultaneous processing of a secondary task (Levine et al., 2012).

#### Cognitive Profiles of Heavy Media Multitaskers

In recent years, there has been mounting research interest in determining whether digital multitasking is associated with deficits or benefits to various aspects of cognitive control and information processing (Lui and Wong, 2012; Alzahabi and Becker, 2013; Minear et al., 2013; Ralph et al., 2015; Cain et al., 2016; Uncapher et al., 2016). It has been proposed that multitasking disrupts sustained attention, thereby impeding self-regulatory abilities, motivation, memory and learning (Lee et al., 2012; Wei et al., 2012; Wood et al., 2012; Rosen et al., 2013; Stothart et al., 2015; Grieco-Calub et al., 2017; May and Elder, 2018).

Many studies have linked chronic media multitasking behavior to cognitive operation deficits, such as deficits in sustained attention, working memory, long-term memory, impulse response, and inhibitory control (Uncapher et al., 2016; Schutten et al., 2017; Uncapher and Wagner, 2018). For example, an early study by Ophir et al. (2009) tested whether engaging in frequent multitasking could help train the ability to hold items in short term memory, to switch between tasks, and to ignore irrelevant information. Contrary to their expectations, the researchers found that self-reported heavy media multitaskers (HMM) performed worse on a variety of cognitive control tasks, relative to light media multitaskers (LMM). The authors concluded that heavy media multitaskers may differ in attentional- and cognitive-control abilities and have a greater tendency for bottom-up (i.e., automatic and exploratory) processing, compared to LMM.

While these results suggest that frequent media multitasking may negatively interact with top-down cognitive control, taken together, the subsequent literature only partially supports this claim. In a recent replication study, Wiradhany and Nieuwenstein (2017) failed to reproduce the findings by Ophir et al. (2009) which linked chronic media multitasking to cognitive deficits. Several other cross-sectional studies found that heavy media multitasking was not related to behavioral measures of cognitive control (Baumgartner et al., 2014; Cardoso-Leite et al., 2015). Moreover, some studies suggest that there may even be cognitive benefits to frequent media multitasking (Lui and Wong, 2012; Alzahabi and Becker, 2013; Yap and Lim, 2013).

Overall, the evidence suggests that while individuals who multitask heavily with technology tend to *self-report* more attention difficulties, distractibility, and impulsivity (Levine et al., 2007; Bowman et al., 2010; Junco and Cotten, 2012), these subjective assessments do not necessarily align with objective performance-based measures (Junco, 2012; Levine et al., 2013; Baumgartner et al., 2014). For example, higher levels of multitasking seem to be related to self-assessed everyday attention lapses, but unrelated to performance-based measures in the domains of sustained-attention, working memory, interference management, task-goal management, and inhibitory control (for reviews see: Van Der Schuur et al., 2015; Uncapher et al., 2017; Wiradhany and Nieuwenstein, 2017).

The reason for this inconsistency might be that performance-based and self-report assessments measure different aspects of cognition (for a discussion see: Toplak et al., 2013). Moreover, these results suggest that heavy media multitaskers are not necessarily *less able* to control and sustain their attention, but rather, that heavy multitaskers may *choose* to engage differently with their environment (Ralph et al., 2015). This may reflect individual differences in thresholds for motivation and engagement, rather than attention *per se*. Indeed, high levels of media multitasking has also been linked to greater impulsivity (Sanbonmatsu et al., 2013), greater delay discounting (Wilmer and Chein, 2016), and a preference for speed at the expense of accuracy on cognitive assessments (Minear et al., 2013).

#### Media Multitasking and Learning

The majority of studies show that multitasking with media devices during learning is negatively related to three main areas of academic performance: academic outcomes, academic attitudes and behaviors, and perceived learning (Van Der Schuur et al., 2015). This may be attributed to the fact that media multitasking may displace the amount of time dedicated to academic activities (Fox et al., 2009), or that media multitasking may limit the amount of attention available for the simultaneous processing of academic content (Junco and Cotten, 2012). However, because few studies have looked at the precise cognitive mechanisms underlying screen-based multitasking while learning, it is not yet possible to discern which of these two hypotheses is most plausible.

In the academic setting, numerous studies have reported small to moderate negative associations between multitasking with mobile devices and various aspects of academic performance (Kuznekoff and Titsworth, 2013; Lepp et al., 2014; Chen and Yan, 2016; Dempsey et al., 2019; Baert et al., 2020). For example, one small classroom study by Bowman et al. (2010) reported that students who instant messaged (IM) while reading a passage took significantly longer to read the text, even after accounting for the time actually spent IMing. However, the authors also found that reading comprehension scores were not affected by the condition, indicating that multitasking may decrease the degree of efficiency required for achieving the same level of performance on a task, while not necessarily affecting accuracy.

Given the growing use of laptops in educational contexts, many cross-sectional studies have also investigated whether using laptops in class may impede learning. Results from several studies suggest that performance deficits associated with increased multitasking behavior may be particularly prevalent during off-task (i.e., non-academic) usage (Hembrooke and Gay, 2003; Fried, 2008). Indeed, using social media while learning has been shown to impair comprehension and test performance (Kirschner and Karpinski, 2010; Junco and Cotten, 2012; Sana et al., 2013). Background media, like television, has also been shown to reduce the quantity and quality of concurrent activities, including homework and sustained play, which is integral for cognitive and socio-emotional development for young children (Kirkorian et al., 2009; Adler and Benbunan-Fich, 2013). However, although media multitasking in the context of learning seems to be cross-sectionally related to academic achievement, more research longitudinal research does not find support for an association between multitasking and subsequent academic achievement (van der Schuur et al., 2020).

It should also be noted that the majority of studies investigating the effects of multitasking on academic performance have been conducted using cross-sectional designs, with small convenience samples of limited populations and rely considerably on self-report. The wide variability in measurement and task design (e.g., type of multitasking activity and context) may account for some of the conflicting findings within the literature. Moreover, course grades are often used as proxies to infer the effects of media multitasking on attention or cognitive control, but such studies generally do not measure these constructs directly. It, therefore, remains difficult to interpret the mechanisms through which the observed effects may be occurring in the short-term, and what specific cognitive functions may be implicated in the long term.

#### The Who: Individual Differences in Multitasking Outcomes

There is some evidence that individual differences in cognitive capacity and neural profiles (Lehle and Hübner, 2009; Miller et al., 2009; Reissland and Manzey, 2016; van der Schuur et al., 2020), and functional maturity (Cepeda et al., 2001; Maquestiaux et al., 2004) may moderate the relationship between of multitasking and cognition. For example, age-related improvements in task-switching ability (Reimers and Maylor, 2005) indicate that young children may suffer from more information loss and executive control deficits while engaging in more than one task simultaneously. To date, however, the study of individual differences such as age, gender, socioeconomic background and dispositional moderators in the area of *digital* multitasking has largely been ignored.

It should also be noted that many of the findings on the detrimental effects of multitasking are achieved in controlled experimental settings and focus on very narrow measures of cognitive performance (May and Elder, 2018). From this perspective, the long-term implications and higher-order benefits derived from multitasking with digital technology may be quite different from the immediate effects reported in short-term studies. Similarly, individual differences in thresholds for motivation and engagement, or preference for highly stimulating environments may account for some of the observed variance in multitasking outcomes (Ralph et al., 2015).

#### The What, When, and How: The Importance of Multitasking Quality and Context

The ease of multitasking may be affected by the amount (quantity) of cognitive demands, but also by the type (quality) of cognitive resources required for the task. As such, different multitasking scenarios will produce varying degrees of cognitive load. There is growing evidence that some tasks are more easily combined than others, and studies have shown that people seem to have a natural preference for task combinations that do not overtax their cognitive capacity (Jeong and Fishbein, 2007; Carrier et al., 2009; Wiradhany and Baumgartner, 2019). For example, individuals tend to multitask while listening to music, watching television, or eating, but less so while playing video games or having phone conversations (Voorveld and van der Goot, 2013; Van Der Schuur et al., 2015).

Experimental studies, however, tend to study multitasking effects with tasks that are not easily combined. This calls into question the validity of available evidence and the degree to which it may reflect digital multitasking behavior in the real world. Indeed, many interactive technologies, such as smartphones and laptops, are designed as multitasking facilitators and encourage maximum user efficiency (Pea et al., 2012; Hwang et al., 2014). During online search, for example, it is quite easy to open several windows simultaneously, to switch between different pages, and even shift to a completely different online task while waiting for a document to download. This kind of task-switching behavior may facilitate greater information processing and enhance cognitive efficiency in the long term (Hwang et al., 2014; Wang et al., 2015). Differences between experimental and real-world multitasking conditions could also explain why media multitasking does not seem to be related with academic achievement longitudinally (van der Schuur et al., 2020).

While few studies have directly assessed the facilitating role of media multitasking, one meta-analysis found that user control, task relevance and contiguity (i.e., the physical distance between tasks) moderated the effects of multitasking on cognitive performance (Jeong and Hwang, 2016). When user control is high, individuals are more easily able to adjust the task-switching speed and pace of content to decrease their cognitive load. Additionally, the cognitive load of multitasking is lesser when two tasks are related and physically near, particularly for visual tasks (i.e., which are displayed on a single medium). Therefore, drawing conclusions about the effects of digital multitasking as a whole may be over-simplistic. Outcomes likely differ based on the degree of cognitive and motor resources required for each individual task (i.e., active vs passive engagement); how closely they relate to one another (conceptually and physically); and how easily they are combined.

Cognitive outcomes also likely depend on the specific measures of cognitive performance, the context of use, and the digital devices being examined (Wang and Tchernev, 2012; Jeong and Hwang, 2015; Wang et al., 2015). Existing studies on digital multitasking, however, often examine only one particular technology use-case (e.g., social media, television, or IM). This narrow focus, however, does not accurately reflect the complex media-use patterns of young people, who often engage with multiple digital technologies simultaneously (Lee et al., 2012). Moreover, existing studies have only examined the impact of contextual factors related to academic settings, but there is a lack of research on whether engaging in screen-based multitasking may enhance or disrupt other areas of daily life.

#### Digital Multitasking Summary

Taken together, the weight of the evidence does not conclusively demonstrate that digital multitasking impairs attention and cognitive control in a global or persistent manner. Heavy media multitasking seems to be negatively related to subjective measures of attention in everyday life, but unrelated to objective measures of sustained-attention and cognitive control (Van Der Schuur et al., 2015; Uncapher et al., 2017). These results may reflect individual differences in motivation and engagement, rather than attention *per se* (Baumgartner et al., 2014), suggesting a potential bidirectional link between multitasking frequency and cognition. However, there remains a paucity of longitudinal research to establish causality. Multitasking with digital devices while learning has also been shown to impede comprehension, recall, and academic performance (Van Der Schuur et al., 2015). However, this may be due to the reduction of time dedicated to academic activities (Fox et al., 2009), rather than the disruption of sustained attention. Moreover, there is some indication that individual differences in neurocognitive profile (Miller et al., 2009), disposition (Ralph et al., 2015), and age (Reimers and Maylor, 2005) may moderate the relationship between task-switching and cognition. However, the study of individual differences has largely been ignored in the digital multitasking literature. Finally, there is growing evidence that some tasks are more easily combined than others, thereby facilitating greater information processing (Wang et al., 2015). Future research should focus on examining contextual moderators to discern when and how digital multitasking may be beneficial.

## Discussion

The rapid evolution of the technological landscape – the devices themselves, the nature of the content, and how they are used – has made it increasingly difficult to discern how digital devices might interact with cognition in a way that is relevant to the present generation of media users (Marsh, 2014). This difficulty is determined by changes in technologies as well as by changes in the profile of users, who are more diverse than ever before. The existing literature often reflects outdated digital devices and patterns of use, while also failing to take into account the nuances of how users engage with more modern ‘connected devices’. This has created a lag in the scientific literature and also in the guidelines which they serve to inform (Straker et al., 2018), so much so that often recommendations based on ‘screen time’ do not reflect the nuanced picture painted by the scientific literature (American Academy of Pediatrics, 2016; Rütten and Preifer, 2016; Ponti et al., 2017). However, there is still much value to be gained from the existing literature if adequately put in context. In particular, the building blocks that have been identified can provide useful insight for evaluating how newer and more sophisticated forms of digital media might interact with human cognition.

A central theme emerges from the existing literature: different features of technology and different types of use contribute to cognitive outcomes in different ways. Technology should not be viewed as a homogenous or neutral stimulus (Bavelier et al., 2010; Subrahmanyam and Renukarya, 2015). Similarly, individual differences such as age, cognitive ability, prior experience, interest and motivation influence how, when and why individuals use technology, as well as how much benefit or harm can be derived from its use (Corbetta and Shulman, 2002; Azevedo and Hadwin, 2005; Moos and Azevedo, 2009). Not all individuals will be equally affected by technology, just as not all technologies affect cognition in a global and persistent manner. These results highlight the limitations of generalizing across different screen-based activities when discussing the cognitive implications of digital technologies. Taken together, the existing literature suggests that the cognitive implications of digital technology use are moderated by three related factors: timing and age considerations, the degree of user engagement and control; and the alternative use of time.

### Timing and Age Considerations

The literature suggests that both the frequency and cumulative time spent engaging with digital media may matter. Overall, there is little support for a negative long-term association between digital technology use in childhood in *moderate* amounts and long-term cognitive deficits (Foster and Watkins, 2010; Pujol et al., 2016). However, the evidence also indicates that the greater the cumulative time spent engaging with digital media (i.e., age of initial exposure), the greater the cognitive implications, both good and bad (Hartanto et al., 2016). For example, cross-sectional studies suggest that individuals who begin playing video games at earlier ages perform better on various measures of attention, compared to those who began playing at later ages (Dye et al., 2009b; Latham et al., 2013). As such, the common operationalisation of technology use based on the frequency of weekly screen-time (Bavelier et al., 2012b), without accounting for lifelong cumulative exposure and age of initial exposure, may not adequately capture these crucial timing considerations (Hartanto et al., 2016).

The fact that portable devices allow individuals to engage with digital applications anytime anywhere increases the frequency of use and overall amounts of time spent using digital technologies, leading to an increase in the number of children engaging in excessive use from younger ages (Siibak and Nevski, 2019; Ofcom, 2020). New portable devices support applications that limit the amount of time individuals engage with them, that can be set up either by children themselves or by their parents and carers. Installing such applications or implementing other strategies aimed at moderating access to digital media can help mitigate excessive digital media use during development.

The literature also indicates that there are critical age effects in the way children engage with digital technologies. This is because different components of cognition develop at different rates and are more or less plastic across different stages of development (Rueda et al., 2005). For example, certain features of television programming, like fast-pace and non-normative stimulation, may overtax infants’ cognitive resources and encourage bottom-up processing in the short-term (Valkenburg and Vroone, 2004; Goodrich et al., 2009; Lillard and Peterson, 2011). However, these same features may not be problematic for older children, and can even be beneficial to training specific aspects of cognition if presented through interactive mediums, like video games (Pempek et al., 2010; Strobach et al., 2012). Similarly, age-related improvements in cognitive control (Reimers and Maylor, 2005) indicate that young children may suffer from more information loss and executive control deficits while engaging in more than one task simultaneously. This is supported by evidence that even exposure to background television can disrupt sustained-play and reduce the quality and quantity of parent-child interactions, which is critical for language acquisition and the development of cognitive and social skills (Schmidt et al., 2008; Christakis et al., 2009; Barr et al., 2010; Pempek et al., 2014). Therefore, while the literature does not conclusively demonstrate that childhood exposure to technology impacts attention and cognitive control, there is some indication that exposure may not be beneficial for infants whose cognitive faculties may not be sufficiently developed to properly engage with digital media.

Mirroring increases in the amount of time children spend accessing digital media, in recent years there has been an increase in the number of children who access digital technologies at a very early age and who do so for extended amounts of time (Ofcom, 2020). Efforts should be made to ensure that very young children are not exposed to digital media or spend only limited periods of time engaging in digital applications, rather than preventing all children, including older children from engaging with such media.

### The Degree of User Engagement and User Control

The degree of user engagement during digital technology use is also an important factor to consider in moderating cognitive outcomes. We define active user engagement as the ability to exert top-down voluntary attentional (and motor) control over digital media, rather than passive (bottom-up) information processing. For example, although mostly based on cross-sectional data, the literature suggests that there may be certain cognitive training benefits to playing video games that are goal-oriented and highly interactive, particularly in older age groups whose cognitive and motor faculties are sufficiently developed to actively engage with such games (Subrahmanyam and Renukarya, 2015). Therefore, the degree of interactive engagement during digital media use may moderate long-term cognitive outcomes, such as skill acquisition. However, as young children are unable to properly engage with complex digital media (e.g., action video games) which are often the focus of academic research, most existing studies so far have focused on adolescents and adults.

The ability to control the content and speed at which digital media is consumed also matters. When user control is high, individuals are more easily able to adjust the pace of content to decrease the associated cognitive load. For example, infants may learn more readily from touch screen devices than through traditional television screens (Kirkorian et al., 2016). Touch screen devices are interactive and therefore allow children to physically manipulate and control the pace and form of content, which may increase engagement and learning (Neumann, 2018). Similarly, many interactive technologies, such as smartphones and laptops, are designed as multitasking facilitators by allowing users to control the incoming flow of information (Pea et al., 2012; Hwang et al., 2014). This kind of digital media use may facilitate greater information processing and enhance cognitive efficiency in the long term (Jeong and Hwang, 2015; Wang et al., 2015). Therefore, as digital technologies become more sophisticated and allow for more immersive and interactive experiences, the higher-order cognitive benefits of digital technologies may become more pronounced. Parents and education professionals can encourage children to adjust the pace of the content they are exposed to, empowering them to maximize the cognitive benefits of modern technologies and reduce some of the potential pitfalls associated with their uncritical use.

### The Alternative Use of Time

The literature suggests that the long-term implications of digital technology use depend on the type of activities it displaces. This is relevant for understanding why results across the existing literature can be highly heterogeneous. For example, the fact that media multitasking seems to be unrelated to objective measures of cognitive control (Van Der Schuur et al., 2015) suggests that related performance deficits may be due to the fact that media multitasking displaces the amount of time dedicated to secondary tasks (e.g., learning) (Fox et al., 2009). In other words, media multitasking may decrease the degree of efficiency required for a task, while not necessarily affecting cognitive faculties directly. This suggests that the amount of time displaced from secondary tasks is a more robust indicator of the media multitasking effects than objective measures of cognition.

Parenting style and family environment also seem to moderate the relationship between digital technology and cognitive outcomes by determining the value of alternative uses of children’s time and the type of content being accessed. For example, as children develop the ability to comprehend, and therefore, *learn from* television content (Anderson and Hanson, 2010), the long-term implications of educational programming differ depending on whether the alternative uses of children’s time would be devoted to more enriching activities, such as learning or high-quality interaction with parents (Linebarger et al., 2014). Educational technologies, such as game-based learning may, therefore, be particularly beneficial for children from underprivileged households and can help bridge early academic gaps between different socio-economic backgrounds.

Parents, carers, and education professionals should consider working with children to ensure that their digital media use does not displace activities that are associated with their immediate but also long-term health, well-being and cognitive control. Having dedicated time for high-quality parent-child interactions, for engaging in physical exercise and home study can ensure that digital media use does not displace but, rather, complements other enriching ways in which children spend their free time.

### Research Gaps and Future Directions

Although the current review has identified several key findings regarding the cognitive implications of digital technology use among youth, there remain important research gaps. To further advance the field, we propose four directions for future research: examining causality; establishing a theoretical foundation; identifying individual and contextual differences; and improving methods and measurement.

#### Examining Causality

The lack of longitudinal research, particularly in the video-game and multitasking literature, makes it difficult to establish causal direction of the relationship between digital technology use and cognitive function. Although it is typically proposed that digital technologies lead to deficits in attention and cognitive control, the reverse could also be true. For example, it is possible that children with more attention difficulties simply prefer to watch more television due to their cognitive-behavioral dispositions (e.g., higher thresholds for engagement and a preference for more stimulating environments) (Beyens et al., 2018). Similarly, children who find it difficult to sustain attention on a single task may be more inclined to engage in frequent multitasking with their digital devices (Ralph et al., 2015). Indeed, the available evidence suggests that there may be a bidirectional link between technology use and cognition (Gentile et al., 2012; Baumgartner et al., 2014). In other words, individuals may be more drawn to digital technologies that suit their own abilities and preferences. However, it is not currently possible to establish causality given the nature of the data examined. Additional longitudinal and experimental research incorporating individual differences, is needed to address the directionality of the relationship between digital media use and cognition.

#### Establishing a Theoretical Foundation

The review of the existing literature reveals a field of enquiry that lacks a comprehensive theoretical framework that could account for a rapidly evolving technological and social landscape and how this interacts with biological and neurological processes involved in cognitive development. Because of this, the possible cognitive mechanisms underlying the relationship between digital technology use and cognition remain unclear. Without a mechanistic account it remains difficult to say whether multitasking with digital devices is markedly different from multitasking with non-digital tasks, or whether viewing video content through portable media significantly differs from viewing the same content through television sets when considering the effects such viewing has on attention and cognitive control. Therefore, future research should focus on identifying the exact mechanisms through which digital technologies may be interacting with cognition across development.

A clear theoretical foundation would also allow the field to advance by merging the current fragmented literature and lessen researchers’ need to re-evaluate mode-of-delivery effects every time a new digital technology becomes available. Such a framework should focus on incorporating how factors like formal features, content, context, individual and social factors moderate how much benefit or harm can be derived from using digital technology. This would allow emerging technologies to be evaluated against an existing evidence base, rather than disregarding past research altogether and attempting to answer the same basic questions that have already been answered in the past (Orben, 2020). It would also allow the field to move past the prevailing causational viewpoint, which assumes that all individuals are equally affected by the new technology (Grimes et al., 2008).

#### Identifying Individual and Contextual Differences

Although some researchers suggest that individual differences in cognitive ability, motivation, engagement, and interest may moderate how, when and why individuals use technology, as well as how much benefit or harm can be derived from its use (Moos and Azevedo, 2009; Przybylski and Wang, 2016), studies investigating individual differences remain scarce. For example, in the gaming literature, there still remains much debate regarding whether the reported benefits of game-play are due to pre-existing group differences between gamers and non-gamers (Powers et al., 2013). Moreover, video game research conducted on younger children is only beginning to emerge, which makes it difficult to assess how interactive technologies may differentially affect children at different stages of development. Similarly, the study of individual differences in the area of media multitasking has largely been ignored. To further develop our understanding of how digital media may be interacting with cognition, future research should focus on identifying which individuals may be more susceptible to the effects of digital media by investigating demographic (e.g., age, gender), dispositional (e.g., engagement, motivation), and cognitive (e.g., ability, experience) moderators.

Contextual differences may also moderate the cognitive implications of engaging with digital media. However, while the television literature examines some contextual moderators (e.g., family, academic), there is a paucity of research on whether these factors moderate long term cognitive outcomes associated with using other digital media, such as video games. This is particularly surprising given the prevalent enthusiasm for game-based learning in the context of formal education and the theorized potential of interactive technologies to support learning in children who struggle at school (Schmidt and Vandewater, 2008). Conversely, the multitasking literature examines contextual factors almost exclusively related to academic settings, but there is a lack of research on whether engaging in digital multitasking may enhance or disrupt other areas of daily life. Moreover, existing studies on digital multitasking and cognitive performance often include only one particular type of technology use (e.g., social media, television, IM) (Van Der Schuur et al., 2015). This narrow focus, however, does not accurately reflect the complex media-use patterns of young people, who often engage with multiple digital technologies simultaneously in a way that might facilitate information processing (Lee et al., 2012; Wiradhany and Baumgartner, 2019). Therefore, future research should focus on investigating which types of media use are particularly beneficial and which are disruptive.

#### Improving Methods and Measurement

Although concerns regarding screen-based media primarily focus on children, few experimental studies have actually been conducted on children. Moreover, the literature is characterized by small convenience samples, small effect sizes, and questionable tools for measuring digital technology use patterns. In the short term, the field would be aided by more high-powered experimental research, with larger samples of diverse populations and more robust statistical methodology which takes into account individual factors, social demographics, and technology use histories. Moreover, when possible, studies should aim to include multiple measures of the same cognitive process and emphasize which precise outcome variables are being assessed (i.e., which subdomains of cognition), why they have been chosen, and how they are measured.

The overreliance on self-report should also be noted as a limitation in much of the existing literature. In particular, the accuracy and validity of self-reported technology use is often quite low, whereby both over-and under-reporting is commonplace (Moreno et al., 2012; Junco, 2013; Scharkow, 2016). Furthermore, pre-tested and validated scales that are commonly used in psychology studies are often abbreviated or altered for the purpose of large-scale surveys to reduce participant burden, which generally diminishes the quality of the measures and limits the ability to infer from the resulting data (Livingstone et al., 2015; U. S. Department of Health and Human Services, 2015). Moreover, there are important generational changes in the way users use digital technologies, which are often overlooked in the literature. Media-use questionnaires should therefore reflect the complexity of modern-day media devices and use-patterns, such as content creation, social media, and multitasking behaviors. The extent to which media consumption is measured by metrics like weekly screen time should also be reviewed.

Finally, the overly negative focus and sensationalist claims in the digital technology literature tend to garner significant interest in popular media and social discourse (Ferguson, 2007; Elson and Ferguson, 2014; Granic et al., 2014). As outlined in the present review, the long-term and higher-order implications of using digital technologies may be quite different from the immediate effects observed in short-term experimental contexts. Future studies should aim to contextualize their findings against the backdrop of the larger evidence-base in order to provide a more nuanced view of the effects of digital technology on youths’ cognitive function.

## Author Contributions

MV conceived the study, reviewed the literature, and drafted the manuscript. FB conceived the study and drafted the manuscript. Both authors contributed to the article and approved the submitted version.

## Conflict of Interest

The authors declare that the research was conducted in the absence of any commercial or financial relationships that could be construed as a potential conflict of interest.
